# CD64 as novel molecular imaging marker for the characterization of synovitis in rheumatoid arthritis

**DOI:** 10.1186/s13075-023-03147-y

**Published:** 2023-08-31

**Authors:** Wessel F. Theeuwes, Irene Di Ceglie, Daphne N. Dorst, Arjen B. Blom, Desiree L. Bos, Thomas Vogl, Sander W. Tas, Pilar Jimenez-Royo, Mats Bergstrom, Matthew Cleveland, Peter M. van der Kraan, Peter Laverman, Marije I. Koenders, Peter L. van Lent, Martijn H. J. van den Bosch

**Affiliations:** 1 Department of Experimental Rheumatology, Radboud University Medical Center, Nijmegen, The Netherlands; 2 Department of Medical Imaging, Radboud University Medical Center, Nijmegen, The Netherlands; 3 Institute of Immunology, University of Münster, Münster, Germany; 4 Department of Clinical Immunology & Rheumatology, Amsterdam Rheumatology and Immunology Centre, Academic Medical Centre, University of Amsterdam, Amsterdam, The Netherlands; 5 Research and Development, GlaxoSmithKline, Stevenage, UK; 6 Bioimaging, In Vitro/In Vivo Translation (IVIVT), GlaxoSmithKline, Stevenage, UK

**Keywords:** CD64, Rheumatoid arthritis, Macrophage, Molecular imaging, Preclinical models

## Abstract

**Background:**

Rheumatoid arthritis (RA) is one of the most prevalent and debilitating joint diseases worldwide. RA is characterized by synovial inflammation (synovitis), which is linked to the development of joint destruction. Magnetic resonance imaging and ultrasonography are widely being used to detect the presence and extent of synovitis. However, these techniques do not reveal the activation status of inflammatory cells such as macrophages that play a crucial role in synovitis and express CD64 (Fc gamma receptor (FcγR)I) which is considered as macrophage activation marker.

**Objectives:**

We aimed to investigate CD64 expression and its correlation with pro-inflammatory cytokines and pro-damaging factors in human-derived RA synovium. Furthermore, we aimed to set up a molecular imaging modality using a radiolabeled CD64-specific antibody as a novel imaging tracer that could be used to determine the extent and phenotype of synovitis using optical and nuclear imaging.

**Methods:**

First, we investigated CD64 expression in synovium of early- and late-stage RA patients and studied its correlation with the expression of pro-inflammatory and tissue-damaging factors. Next, we conjugated an anti-CD64 antibody with IRDye 800CW and diethylenetriamine penta-acetic acid (DTPA; used for ^111^In labeling) and tested its binding on cultured THP1 cells, ex vivo RA synovium explants and its imaging potential in SCID mice implanted with human RA synovium explants obtained from RA patients who underwent total joint replacement.

**Results:**

We showed that CD64 is expressed in synovium of early and late-stage RA patients and that *FCGR1A*/CD64 expression is strongly correlated with factors known to be involved in RA progression. Combined, this makes CD64 a useful marker for imaging the extent and phenotype of synovitis. We reported higher binding of the [^111^In]In-DTPA-IRDye 800CW anti-CD64 antibody to in vitro cultured THP1 monocytes and ex vivo RA synovium compared to isotype control. In human RA synovial explants implanted in SCID mice, the ratio of uptake of the antibody in synovium over blood was significantly higher when injected with anti-CD64 compared to isotype and injecting an excess of unlabeled antibody significantly reduced the antibody-binding associated signal, both indicating specific receptor binding.

**Conclusion:**

Taken together, we successfully developed an optical and nuclear imaging modality to detect CD64 in human RA synovium in vivo.

**Supplementary Information:**

The online version contains supplementary material available at 10.1186/s13075-023-03147-y.

## Introduction

Rheumatoid arthritis (RA) is one of the most prevalent and debilitating joint diseases worldwide [1]. It is characterized by a strong inflammatory response in the synovial membrane (synovitis) which is linked to the development of joint destruction [2]. In addition to cells of the adaptive immunity (T and B cells), macrophages are abundantly present in the synovial lining and sub-lining of RA patients and play an active role in synovitis [3]. Traditional anatomical imaging modalities such as magnetic resonance imaging (MRI) and ultrasonography are widely used to detect the presence and extent of joint inflammation. These imaging methods, however, do not reveal the activation status of inflammatory cells. There is a clinical need to detect the activation status of immune cells and the damaging potential of synovitis, which vary considerably between patients and strongly influence disease outcome [4]. Therefore, a novel imaging technique that determines these, and that ideally can be used before irreversible joint damage occurs, would be of great benefit for proper diagnosis and to improve the prognosis of RA.

Macrophages play a crucial role in RA synovial inflammation and changes in synovial sublining macrophages can be used to predict the efficacy of antirheumatic treatment [1]. CD64 (Fc gamma receptor (FcγR)I) belongs to the family of activating FcγRs, which are receptors for immunoglobulin (Ig)G-containing immune complexes (IgG-ICs), is mainly expressed on immune cells of myeloid origin. CD64 can be detected on cells in the circulation, synovium, and synovial fluid of RA patients [2–4], and is considered a macrophage activation marker [5]. The expression of CD64 is absent in the synovial tissue of healthy individuals but becomes strongly increased during RA [6]. Previously, it was found that CD64 is crucial in mediating cartilage destruction during IC-mediated RA [7]. Its presence in the synovium and association with synovitis and joint damage in RA make CD64 an interesting imaging marker.

In the present study, we investigated the range of CD64 expression in synovium of early and late-stage RA patients and studied the correlation of its expression and the expression of pro-inflammatory and tissue-damaging factors. To develop an imaging technique for CD64 in vivo, we conjugated an anti-CD64 antibody with IRDye 800CW and diethylenetriamine penta-acetic acid (DTPA; used for ^111^In labeling) and tested its imaging potential in severe combined immunodeficient (SCID) mice implanted with human RA synovium explants [8, 9].

## Materials and methods

### Antibody conjugation and radiolabeling

The commercially available anti-CD64 antibody (Clone 10.1, Labned, 11–644-C100) and mouse lgG1κ isotype antibody (Clone MOPC-21, STEMCELL TECHNOLOGIES) were dialyzed in a Slide-A-Lyzer Dialysis Cassette (20.000 MWCO, Thermo Fisher Scientific) against phosphate-buffered saline (PBS). Thereafter, antibodies were conjugated with infrared (IR)Dye 800CW-NHS (LICOR Bioscience) and S-2-(4-isothiocyanatobenzyl)(ITC)-DTPA (Macrocyclics) by incubating them with IRDye 800CW-NHS and 10% (w/v) 0.1 M NaHCO_3_, pH 8.5 (threefold molar excess of the dye) for 1 h at room temperature (RT). Subsequently, a tenfold molar excess isothiocyanatobenzyl-(ITC)-DTPA and 10% (w/v) 0.1 M NaHCO_3_, pH 9.5 was added and incubated for 1 h at RT. Finally, the mixtures were dialyzed in a Slide-A-Lyzer Dialysis Cassette (cut-off 20.000 MWCO, Thermo Fisher Scientific) against PBS with 1% Chelex (Bio-Rad). The final concentration of the conjugated antibodies was measured spectrophotometrically using the Infinite 200® Pro (Tecan group Ltd.) at 280 nm, correcting for the absorption of IRDye 800CW (at 774 nm). The substitution ratio of the dye reached 1.7 for the CD64 antibody (both batches produced) and 0.7 for the lgG1κ isotype. Labeling with [^111^In]InCl_3_ (Covidien BV, Petten, the Netherlands) was performed as described previously [10]. The labeling efficiency was determined by instant thin-layer chromatography. If the labeling efficiency was below the predefined threshold, the labeled antibody was purified using a Zeba™ Spin Desalting Column (40 K MWCO) (Thermo Fisher). The radiochemical purity was > 90% for in vitro studies and > 95% for in vivo experiments.

### Synovial tissue

Synovium biopsies of early RA patients were collected by routinely performed mini-arthroscopic tissue sampling of a knee joint after informed consent and in accordance with the principles of the Declaration of Helsinki [11]. Early RA patients met the 2010 ACR/EULAR Classification Criteria for RA with active disease (Disease Activity Score evaluated in 28 joints (DAS28) > 3.2). All early RA patients were biologically naïve and had a median disease duration of 6.2 months (interquartile range (IQR) 4.1–8.3). After collection biopsies were immediately stored in OCT. Late RA synovial tissue was perioperatively obtained from RA patients who underwent joint replacement surgery of the hip, knee, shoulder, or elbow. All patients met the 2010 ACR/EULAR Classification Criteria for RA at the time of diagnosis. No exclusion criteria were introduced regarding the treatment received by the patients before or at the time of the surgery, the presence of OA was not excluded with X-rays before surgery. The human biological samples were sourced ethically, and their research use was in accordance with the terms of the informed consents under an IRB/EC-approved protocol. Surgical material was collected in Dulbecco’s modified Eagle’s medium (DMEM) (Thermo Fisher Scientific) supplemented with penicillin/streptomycin. The presence of synovium was macroscopically assessed. All synovial tissue isolated was divided into small biopsies and randomized (to mimic the heterogeneity within joint). Subsequently, the biopsies were stored in OCT for histological analysis, cultured to generate conditioned media, and/or processed for ribonucleic acid (RNA) isolation (all described in more detail in Supplemental methods). Additionally, material collected from some late-stage RA patients was used for ex vivo binding assays or transplanted in SCID mice. For these applications, the macroscopically selected synovium was a priori further analyzed using hematoxylin and eosin (H&E)-stained cryosections. Only material from patients showing histologically active synovitis was used for these downstream applications. A flow chart depicting the collection and analysis procedure of early and late-stage RA material is presented in Supplemental Fig. 1.

### Mice

Female CB17-SCID mice (CB17/lcr-Prkdcscid/Rj) (Janvier Laboratories, France) were group-housed in individually ventilated cages on a 12 h day-night cycle with ad libitum access to standard chow and water. All animal experiments were conducted according to Dutch law and approved by the Dutch Central Authority for Scientific Procedures on Animals (AVD 103002016786 and AVD 10300202010389). All animal studies were ethically reviewed and carried out in accordance with European Directive 2010/63/EEC and the GSK Policy on the Care, Welfare and Treatment of Animals.

### Synovium-SCID model

After assessment of tissue quality on H&E-stained cryosections, inflamed (clinically active synovitis) synovial biopsies (6 mm) were transplanted in anesthetized (inhalation of 2.5% isoflurane with a mix of air and O_2_) CB17-SCID mice. After swabbing the skin with 70% ethanol and shaving the neck, a small incision in the skin between the ears was made. Two synovial biopsies were inserted subcutaneously in the shoulder regions (one on each side). The wound was closed with suture clips. At least 7 days after the synovial transplantation mice were injected with the [^111^In]In-DTPA-IRDye 800CW anti-CD64 antibody, [^111^In]In-DTPA-IRDye 800CW isotype antibody or an excess of unlabeled anti-CD64 antibody at the indicated dose (for single-photon emission computed tomography (SPECT) 10 MBq/mouse and for biodistribution 0.5 MBq/mouse). Mass doses administered are given in the “Results” section.

### In vivo optical imaging, SPECT-CT imaging, and biodistribution after dissection

For SPECT images, mice were scanned under general anesthesia (isoflurane and air) for 1 h in prone position (USPECT/CT, MILabs, Utrecht, the Netherlands). A 1 mm pinhole ultra-high sensitivity mouse collimator was used. The SPECT scans were followed by computed tomography (CT) scans (65 kV, 615 μA). SPECT scans were reconstructed through MILabs software, using 0.2 mm voxel size, 1 iteration, and 16 subsets. For optical imaging, mice or dissected organs were imaged with the IVIS Lumina (Caliper Life Sciences, Hopkinton, MA, USA) with an excitation filter of 745 nm and an emission filter ICG for 1 min acquisition. The biodistribution of the antibodies was determined by weighing organs/tissues of interest and measuring ^111^In using an automated, well-type γ-counter (WIZARD^2^, 2480 Automatic Gamma Counter, Perkin Elmer, USA).

### In vitro binding assay on THP1 cells

THP1 monocytes were cultured in Roswell Park Memorial Institute (RPMI) 1640 medium (Thermo Fisher Scientific) containing 10% FCS and penicillin/streptomycin. To determine antibody binding, 20,000 counts per minute (cpm) [^111^In]In-DTPA-IRDye 800CW anti-CD64 antibody or [^111^In]In-DTPA-IRDye 800CW isotype in 100 μL RPMI supplemented with 0.5% bovine serum albumin (BSA) (corresponding to 0.04 μg/mL of antibodies) were added to 0.5 × 10^6^ cells and incubated for 4 h at 37 °C with 5% CO_2_. The specificity of the binding was evaluated by adding an excess (100 ×) of unlabeled antibody. After washing, the radioactivity bound to the cells was measured with the γ-counter.

### Synovium ex vivo binding assay

Synovium (3 mm diameter) was incubated overnight (20 ± 1 h) with 2 μg/mL of [^111^In]In-DTPA-IRDye 800CW anti-CD64 antibody or [^111^In]In-DTPA-IRDye 800CW isotype in 200 μL RPMI with 0.5% BSA on a shaker at 37 °C with 5% CO_2_. The specificity of the binding was evaluated by adding an excess (100 ×) of unlabeled antibody. Subsequently, the samples were washed for 2.5 h at RT on a roller in PBS, weighed and the amount of radioactivity bound was measured with the γ-counter. Synovium was embedded in OCT, sectioned, mounted and fluorescence was measured with the Odyssey CLx Infrared Imaging System (LICOR Biosciences).

### Statistical analysis

Differences between the two groups were assessed by Student’s unpaired *t*-test. Differences between multiple groups were assessed using One-way ANOVA followed by a Bonferroni multiple comparison posthoc-test with GraphPad Prism (GraphPad software, version 9.3.1). Spearman’s rank correlation coefficients between parameters were determined followed by false discovery rate (FDR) correction (Benjamini and Hochberg, Q = 5%) of the gene expression data (*p*-values ≤ 0.03667 were considered discoveries). Graphs were made using GraphPad and statistical significance of *p* ≤ 0.05, *p* ≤ 0.01, and *p* ≤ 0.001 is indicated by *, **, and ***, respectively.

## Results

### CD64 expression in synovium varies between RA patients

**Fig. 1:**
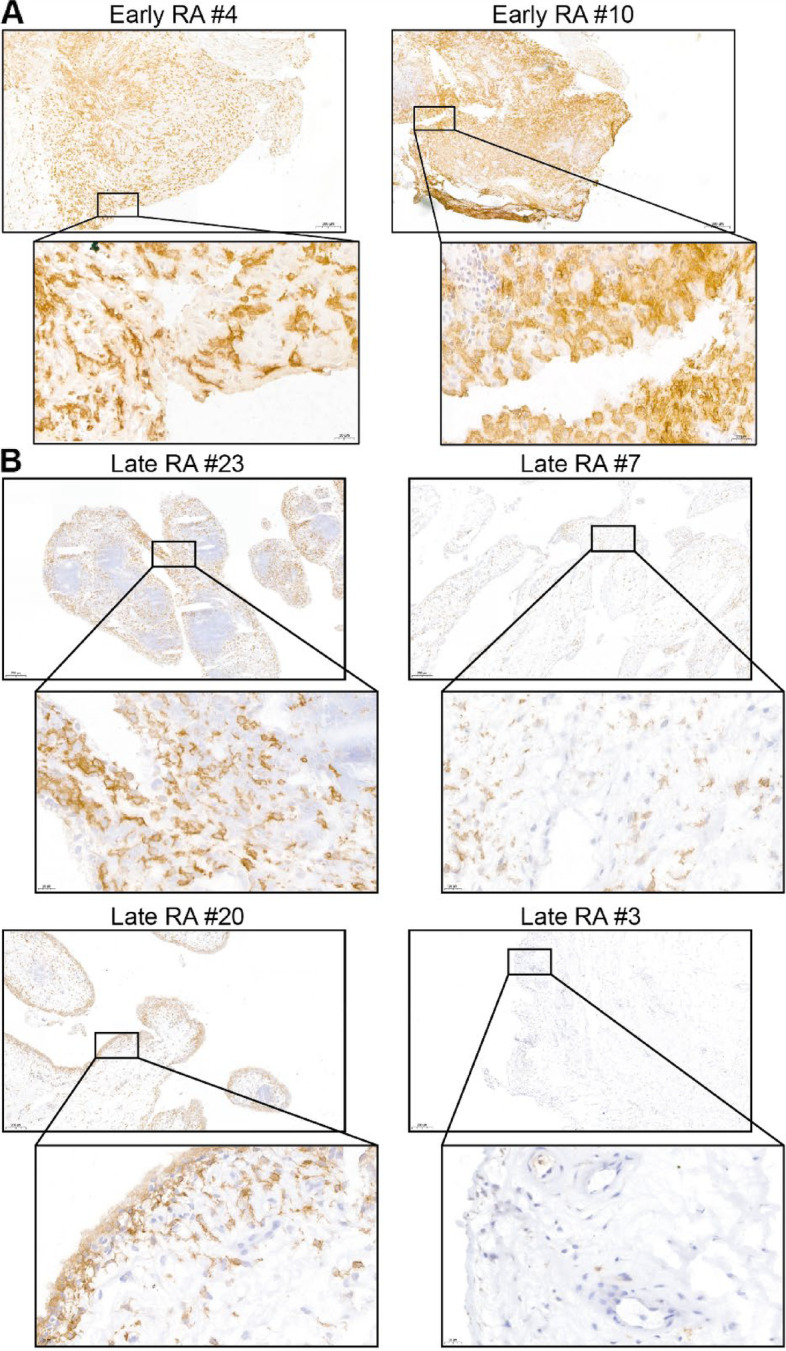
CD64 is expressed in the synovium of early and late-stage RA patients. IHC for CD64 was performed on cryosections of synovium from 10 early RA patients and 24 established RA patients who underwent joint replacement. Representative pictures of synovium from patients with different CD64 expression patterns from **A** early- and **B** end-stage RA synovium are depicted

First, we determined the CD64 expression in synovium obtained from 10 early RA patients and 24 RA patients who underwent joint replacement surgery (late RA), with immunohistochemistry (IHC). This revealed that CD64 is detected in the majority of investigated patients and varies between patients (both in early and late RA). Images of the CD64-staining of early and late-stage RA patients are shown in Fig. 1A and B, respectively. Images from the CD64 IHC of all patients investigated can be found in Supplemental Figs. 2 (early RA) and 3 (late RA). Since the availability of early-stage RA synovium is limited and CD64 was expressed in both early- and late-stage RA synovium, we continued using synovium from established RA in our next experiments.

### CD64 expression correlates with synovial immune cell infiltration

**Fig. 2:**
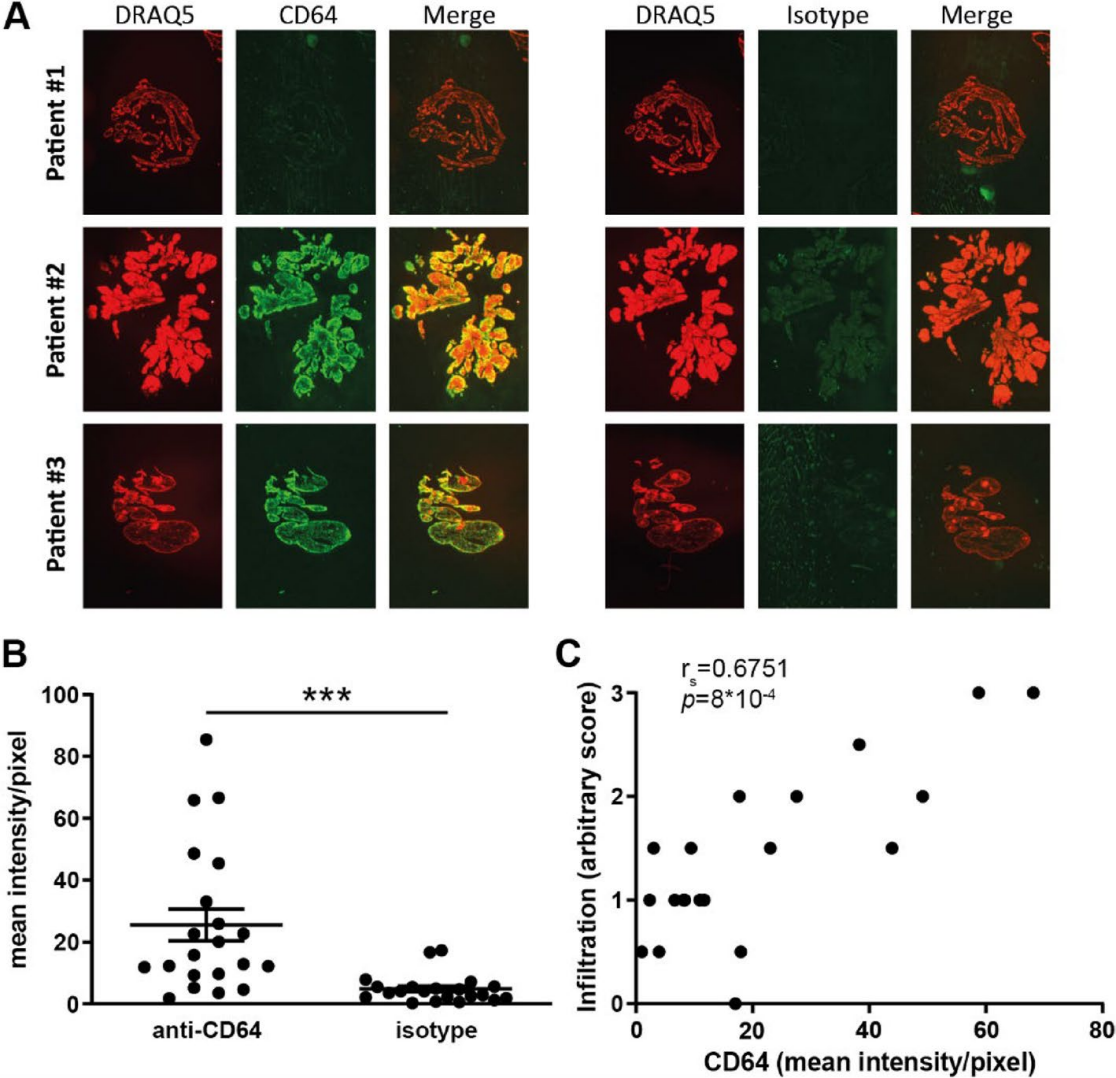
CD64 expression correlates with immune cell infiltration in RA synovium. **A** Examples of fluorescence imaging (Odyssey) of DRAQ5 (nuclei), CD64 (cell membrane), or isotype (on a consecutive section) and merged images from 3 patients. **B** CD64 expression was measured using immunofluorescence. **C** Expression of CD64 correlates with the extent of immune cell infiltration measured with an arbitrary score in the same tissue section. The scatter plot depicts mean ± SEM (**B**) and each data point in the XY graph represents a value of 1 patient (**C**). *n* = 21 late RA patients, *r*_*s*_ = Spearman’s rank correlation coefficient, ***p ≤ 0.001

Next, we quantified fluorescently stained CD64 expression and determined whether its expression correlated with the severity of synovial inflammation (scoring system can be found in Supplemental Fig. 4). We found a wide range of fluorescent intensity of the CD64 staining/pixel, reflecting the variable CD64 expression in RA patients (Fig. 2A, B). This positively correlated with synovial immune cell infiltration (*r*_*s*_ = 0.6751, *p* = 8 × 10^−4^) (Fig. 2C). The fluorescence intensity correlated strongly to the percentage-positive area of the IHC CD64-staining (*r*_*s*_ = 0.8377, *p* = 2 × 10^−6^) (Supplemental Fig. 5).

### Synovial CD64 expression correlates with the expression of pro-inflammatory factors involved in RA pathology

**Fig. 3:**
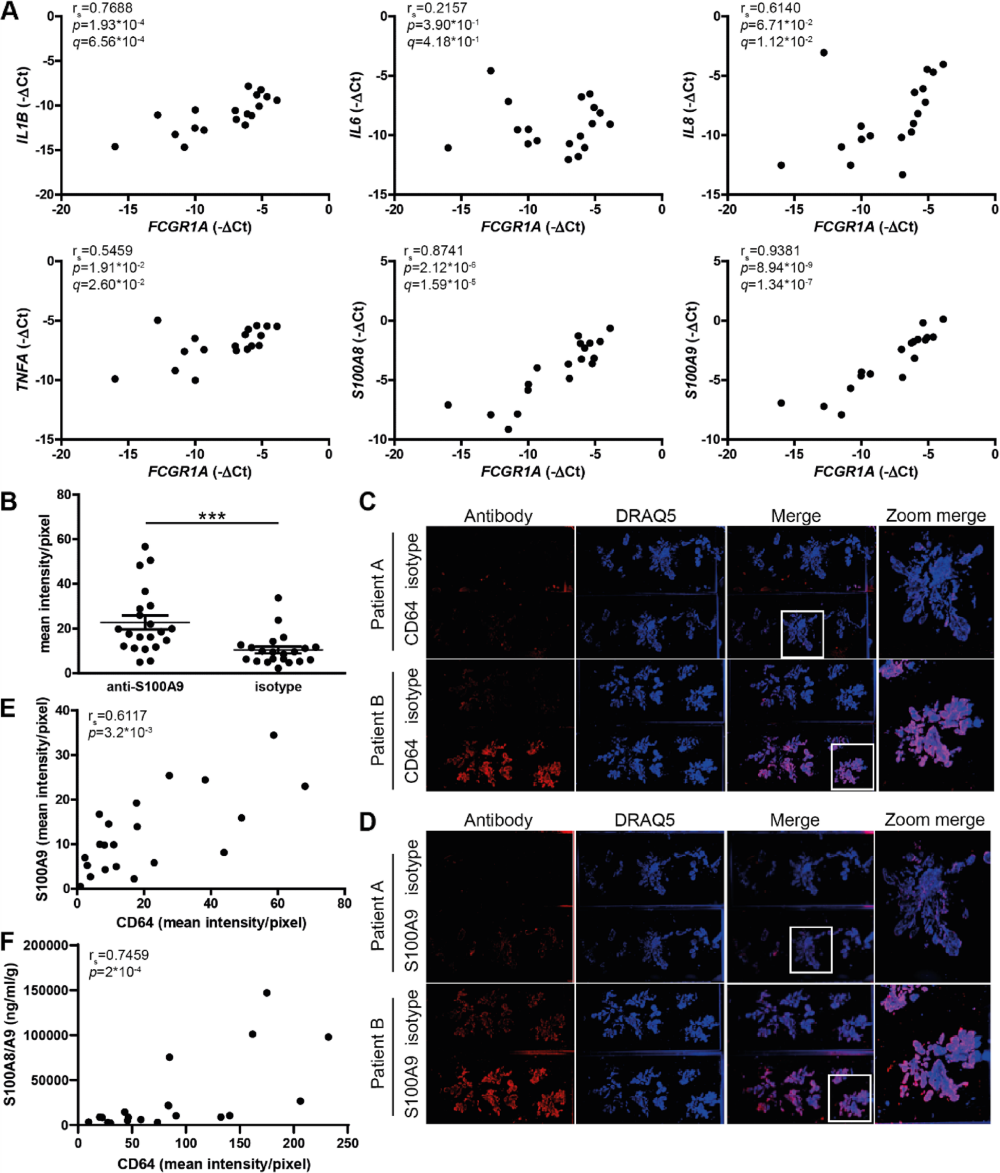
*FCGR1A* gene expression positively correlates with gene expression of pro-inflammatory factors and S100A9 protein levels. **A** Significant positive correlations of *FCGR1A* with *IL1B*, *IL8*, *TNFA*, *S100A8*, and *S100A9* but not with *IL6* were observed. **B** Mean intensity/pixel of S100A9 on cryosection of RA synovium. **C** Representative pictures of CD64 or **D** S100A9 *vs* isotype (scale from red to black) immunofluorescent staining, DRAQ5 (nuclei; scale from blue to black), and merged images (overview and zoomed) from 2 patients. **E** Significant positive correlations of CD64 with S100A9 (corrected for isotype staining in consecutive section) on IHC and **F** released S100A8/A9 in conditioned medium. XY graphs are shown with each data point representing the average value of the samples of 1 RA patient (*n* = 18 for (**A**), *n* = 21 for (**B**, **E**), and *n* = 19 for (**F**), 2–6 synovium pieces/patient). *r*_*s*_ = Spearman’s rank correlation coefficient, *q* = FDR adjusted *p*-value, ***p ≤ 0.001

From a subset of the late RA patients (*n* = 18), we isolated RNA and investigated whether gene expression of *FCGR1A*, the gene encoding CD64, correlated with gene expression of various pro-inflammatory factors well-known to be involved in RA pathology. *FCGR1A* gene expression significantly correlated with the expression of interleukin (*IL)1B* (*r*_*s*_ = 0.7688, *q* = 6.56 × 10^−4^), *IL8* (*r*_*s*_ = 0.6140, *q* = 1.12 × 10^−2^), tumor necrosis factor (*TNF)A* (*r*_*s*_ = 0.5459, *q* = 2.60 × 10^−2^), S100 calcium-binding protein (*S100)A8* (*r*_*s*_ = 0.8741, *q* = 1.59 × 10^−5^), and *S100A9* (*r*_*s*_ = 0.9381, *q* = 1.34 × 10^−7^), while unexpectedly no significant correlation was found with *IL6* (*r*_*s*_ = 0.2157, *p* = 4.18 × 10^−1^) (Fig. 3A).

The alarmin S100A8/A9 is considered as a marker for synovial activation and is a known contributing factor to joint degeneration in RA. We therefore determined the relation between S100A8/A9 and CD64 protein levels. Immunofluorescent staining showed that S100A9 is expressed in the synovium of most patients studied and varies between patients (Fig. 3B). In line with the correlation of *FCGR1A* to *S100A8* and *S100A9* gene expression, S100A9 protein level on immunofluorescence (*r*_*s*_ = 0.6117, *p* = 3.2 × 10^−3^) and the secreted S100A8/A9 levels (*r*_*s*_ = 0.7459, *p* = 2 × 10^−4^) positively and strongly correlated with CD64 expression (Fig. 3C–E).

### Synovial CD64 expression correlates with expression of key markers for pathological processes in the RA joint

**Fig. 4:**
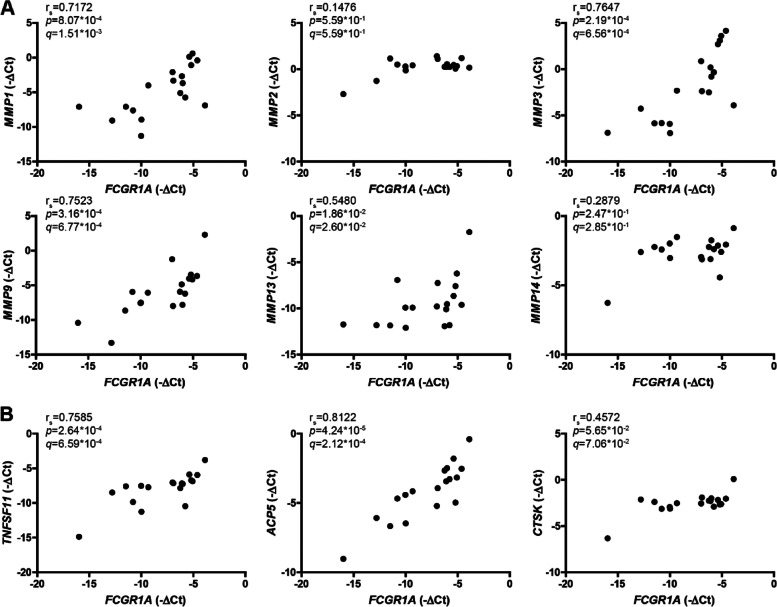
*FCGR1A* positively correlates with matrix-degrading enzymes and markers of bone remodeling in RA synovium. **A** Significant positive correlations of *FCGR1A* gene expression with gene expression of *MMP1*, *MMP3*, *MMP9*, and *MMP13* but not with *MMP2* and *MMP14* were found. **B** *FCGR1A* gene expression positively and significantly correlated with *TNFSF11* and *ACP5* but not with *CTSK*. XY graphs are shown with each data point representing the average value of 1 established RA patient (*n* = 18, 2–6 synovium pieces/patient). *r*_*s*_ = Spearman’s rank correlation coefficient, *q* = FDR adjusted *p*-value

In RA synovium, inflammation often leads to severe cartilage and osteoclast-mediated bone destruction. Importantly, we found that *FCGR1A* gene expression significantly correlated with the mRNA expression of multiple matrix metalloproteases (MMPs) (enzymes well-known for their role in cartilage and bone matrix breakdown), namely *MMP1* (*r*_*s*_ = 0.7172, *q* = 1.51 × 10^−3^), *MMP3* (*r*_*s*_ = 0.7647, *q* = 6.56 × 10^−4^), *MMP9* (*r*_*s*_ = 0.7523, *q* = 6.77 × 10^−4^), and *MMP13* (*r*_*s*_ = 0.5480, *q* = 2.60 × 10^−2^), while no correlation was found with *MMP2* (*r*_*s*_ = 0.1476, *q* = 5.59 × 10^−1^) and *MMP14* (*r*_*s*_ = 0.2879, *q* = 2.85 × 10^−1^) (Fig. 4A). Furthermore, *FCGR1A* expression significantly correlated with TNF Superfamily Member (*TNFSF)11* (*r*_*s*_ = 0.7585, *q* = 6.59 × 10^−4^) (a crucial factor for the induction of osteoclast differentiation) and acid phosphatase (*ACP*)*5* (*r*_*s*_ = 0.8122, *q* = 2.12 × 10^−4^) (early marker of osteoclast differentiation), but not with Cathepsin K (*CTSK)* (*r*_*s*_ = 0.4572, *q* = 7.06 × 10^−2^) (enzyme involved in bone resorption) (Fig. 4B).

### Development of a CD64 imaging modality using a [^111^In]In-DTPA-IRDye 800CW conjugated antibody

**Fig. 5:**
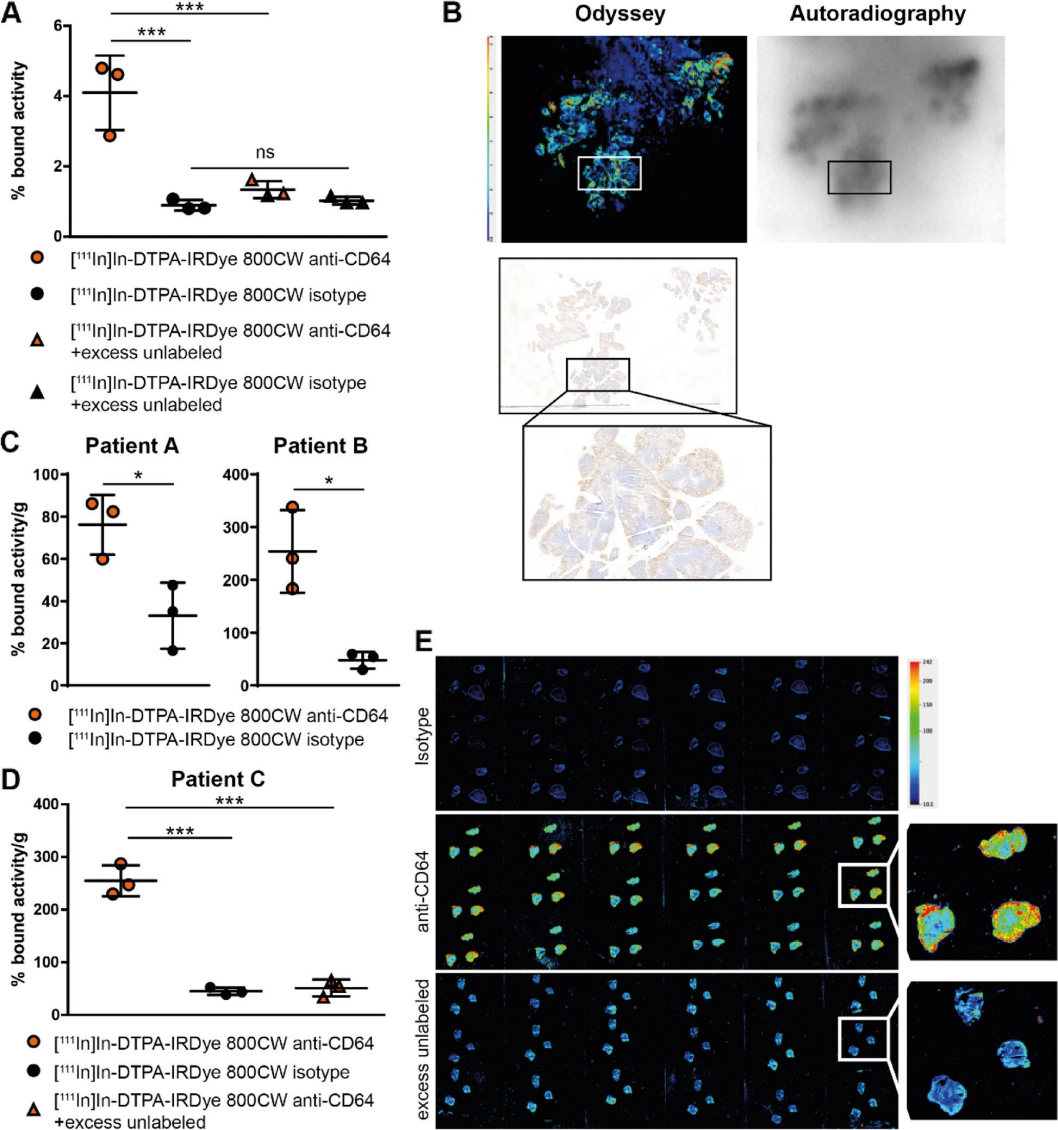
[^111^In]In-DTPA-IRDye 800CW anti-CD64 specifically binds to CD64 in vitro and ex vivo. **A** [^111^In]In-DTPA-IRDye 800CW anti-CD64 showed higher binding to THP1 cells compared to isotype determined using a γ-counter. The signal was blocked by adding an excess of unlabeled anti-CD64 antibody. **B** Odyssey (top left, red (high intensity) to blue/black (low intensity)) and autoradiography (top right) images showed binding of labeled CD64-antibody to RA synovium cryosections. Binding of the radiolabeled anti-CD64 antibody largely overlapped binding of unconjugated CD64 antibody used for IHC (bottom). **C**, **D** [.^111^In]In-DTPA-IRDye 800CW anti-CD64 showed higher binding to human RA synovium explants obtained from 3 RA patients compared to a radiolabeled isotype. **D** An excess of unlabeled antibody decreased the intensity of the anti-CD64 signal detected. **E** Representative images showing the fluorescent signal at various depths of the synovium explant. Scatter plots depicting mean ± SD are shown, *p ≤ 0.05, ***p ≤ 0.001

In order to develop an imaging modality directed against CD64 we used THP1 cells and inflamed RA synovium to set up optical and nuclear imaging. First, we showed that the anti-CD64 antibody gave higher binding to THP1 cells that express CD64 [12] compared to isotype (Fig. 5A). The signal was blocked by adding an excess of unlabeled anti-CD64 antibody, indicating that the signal is specific for antibody-antigen binding. The anti-CD64 antibody was then successfully used to image CD64 on cryosections of inflamed RA synovium. When comparing autoradiography to fluorescent imaging on the same section, the pattern of radioactive signal localized to the same areas as the fluorescent signal, indicating that the radioactive signal is most likely not from free ^111^In. Specificity was further confirmed by IHC of CD64 (gold standard to determine receptor expression localization on tissue sections) using unconjugated CD64 antibody on a consecutive section (Fig. 5B). Moreover, the ^111^In-labeled anti-CD64 antibody showed higher binding to RA synovium explants compared to isotype (Fig. 5C) and competition with an excess of unlabeled anti-CD64 antibody reduced the signal (Fig. 5D). These findings were verified by optical imaging of cryosection from these synovial explants using Odyssey imaging, which allowed visualization of CD64 through several depths of the explants (Fig. 5E).

### Imaging of CD64 in a human synovium-xenograft SCID mouse model

As a final step before testing the radiolabeled anti-CD64 antibody in vivo in SCID mice, we confirmed that the antibody raised against human CD64 did not bind to murine CD64 on bone marrow-derived murine macrophages (Supplemental Fig. 6A), in contrast to a mouse anti-CD64 antibody (Supplemental Fig. 6B). This allows the use of the antibody directed at human CD64 in the human synovium-xenograft SCID model [9].

To determine the optimal antibody dose for imaging, various doses of the radiolabeled anti-CD64 antibody were injected into mice with subcutaneously implanted RA synovium (leftover material from total joint replacement surgery). Biodistribution studies revealed that 3 and 10 μg/mouse of antibody resulted in a higher % injected dose (%ID) of anti-CD64 in the synovial explants compared to 30 μg antibody/mouse (Supplemental Fig. 7A–D). We selected 10 μg/mouse for the next experiments.

**Fig. 6:**
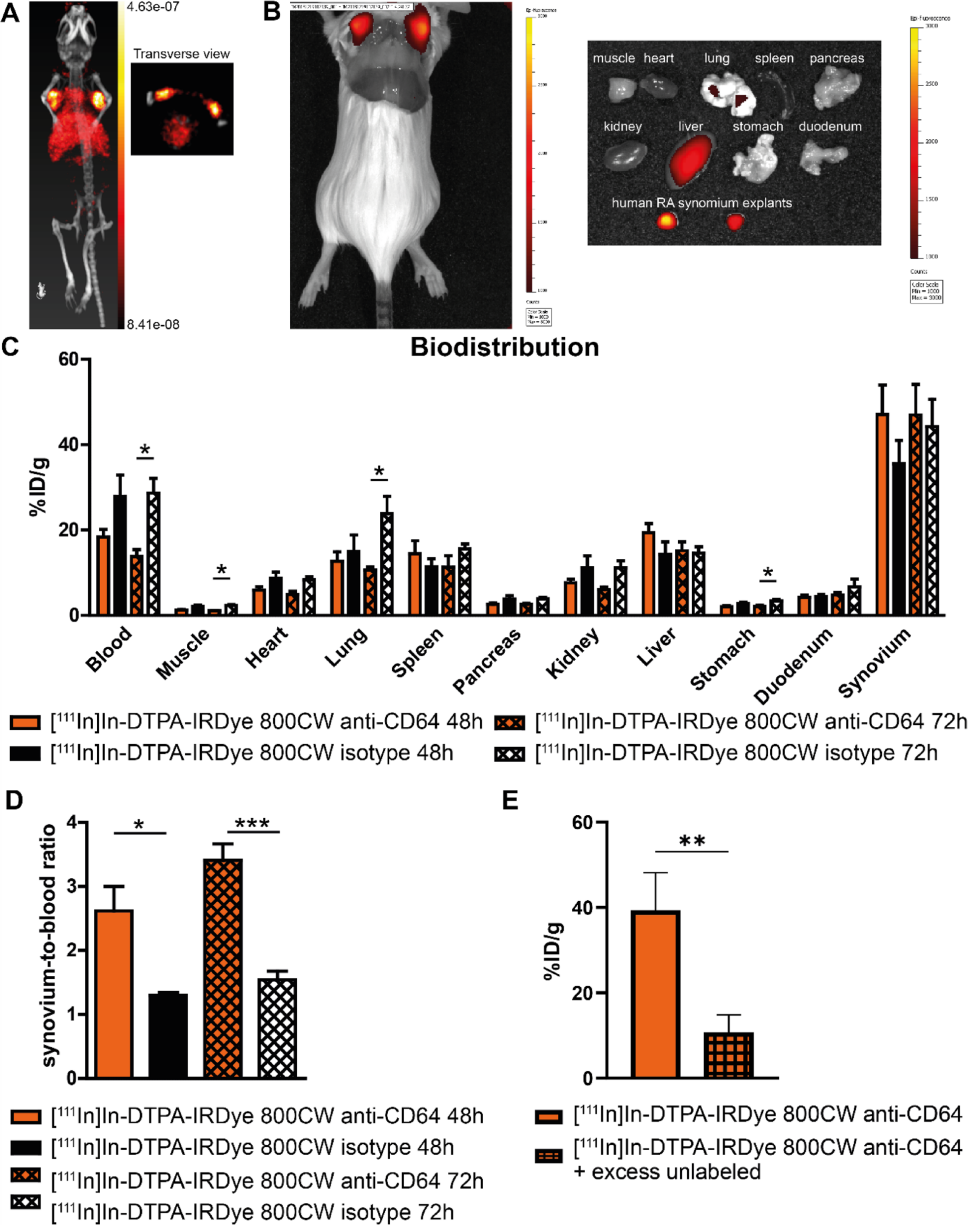
[^111^In]In-DTPA-IRDye 800CW anti-CD64 can be used to image CD64 in implanted inflamed human RA synovium. [^111^In]In-DTPA-IRDye 800CW anti-CD64 or [^111^In]In-DTPA-IRDye 800CW isotype (10 µg/mouse) were injected in SCID mice implanted with RA synovium. **A** Representative USPECT-CT showing the uptake of [^111^In]In-DTPA-IRDye 800CW anti-CD64 in the implanted synovium 48 h after antibody injection. **B** Representative optical images (IVIS) showing the uptake of [^111^In]In-DTPA-IRDye 800CW anti-CD64 in the implanted synovium 48 h after antibody injection (left; whole mice with open skin and Right: organs). **C** Biodistribution of [^111^In]In-DTPA-IRDye 800CW anti-CD64 and [.^111^In]In-DTPA-IRDye 800CW isotype 48 h and 72 h after injection (*n* = 4–5 mice/group). **D** The synovium-to-blood ratio is higher for the anti-CD64 antibody compared to the isotype at 48 h and 72 h. **E** The signal of the anti-CD64 antibody (3 µg/mouse) at 48 h was blocked by co-injecting an excess (190-fold) of unlabeled anti-CD64 antibody (*n* = 7–8 synovium explants/group). Bar graphs depict mean ± SEM. *p ≤ 0.05, **p ≤ 0.01, ***p ≤ 0.001

Representative USPECT-CT (Fig. 6A) and optical IVIS imaging (Fig. 6B) showing the uptake of [^111^In]In-DTPA-IRDye 800CW anti-CD64 in the implanted synovium 48 h after antibody injection. In order to prove the specificity of the signal detected, the biodistribution of the radiolabeled anti-CD64 antibody was compared to a radiolabeled isotype (Fig. 6C). USPECT-CT images from 2 other mice receiving 10 μg or labeled anti-CD64 or isotype are depicted in Supplemental Fig. 8. Although the %ID antibody in the synovium did not differ significantly between anti-CD64 and isotype, the ratio calculated based on %ID of synovium/blood of the anti-CD64 antibody was significantly higher compared to isotype (for 48 h *p* = 0.0193 and for 72 h *p* = 0.0002) (Fig. 6D).

To confirm that the signal measured in the implanted synovium was due to the actual binding of the antibody to synovial cells and not merely due to high vascularization of the tissue, the radiolabeled anti-CD64 antibody was injected with or without an excess of unlabeled anti-CD64. The excess of unlabeled antibody reduced the signal of the anti-CD64 antibody (Fig. 6E), confirming antibody-antigen binding.

## Discussion

In the present study, we showed that CD64 is expressed in synovium of early and established RA patients and that *FCGR1A*/CD64 expression correlates to factors known to be strongly involved in RA progression. Combined, this indicates that CD64 could be a useful marker to image the extent and characteristics of synovitis. Therefore, we developed an optical and nuclear imaging modality directed at CD64 to monitor synovitis in RA synovium. We reported higher binding of the anti-CD64 antibody to in vitro cultured THP1 monocytes and ex vivo RA synovium compared to the isotype. Furthermore, an excess of unlabeled anti-CD64 antibody successfully blocked anti-CD64 antibody binding, indicating specific antibody binding. In human RA synovium explants implanted in SCID mice, the antibody uptake ratio of synovium to blood was significantly higher when injected with anti-CD64 compared to isotype, and injecting an excess of unlabeled antibody reduced the antibody-binding associated signal. Taken together, we successfully developed an optical and nuclear imaging modality to detect CD64 in human RA synovium in vivo.

Important for its potential use as an imaging marker, CD64 is minimally expressed in healthy synovium, while it is expressed in the synovium of many early and end-stage RA patients [6]. Besides expression in the joint, peripheral blood monocytes from early RA patients have higher CD64 expression compared to healthy controls [13], which may partially contribute to high CD64 expression when monocytes infiltrate the joint. CD64 is mainly expressed on myeloid cells and considered an activation marker. CD64 expression on polymorphonuclear neutrophils [14] and monocytes [15] is associated with the amount of inflammation and CD64 is highly expressed on macrophages with a pro-inflammatory phenotype [16]. Importantly, CD64 expression correlated with immune cell infiltration as observed on H&E-staining. Our IHC data confirm the expression of CD64 in the majority of both early and end-stage RA joints, indicating that CD64-imaging can be used to detect synovitis both in early and established RA. However, RA patients with a pauci-immune pathotype probably have low CD64 expression due to the scarcity of myeloid cells. Therefore, it is less likely that CD64 imaging is of added value to determine the damaging potential of the synovitis in these patients.

We reported that *FCGR1A* gene expression is strongly correlated to pro-inflammatory and catabolic factors (MMPs), known to be upregulated during inflammation. These factors play a crucial role in mediating RA-associated processes such as cartilage degeneration and bone remodeling. Besides pro-inflammatory cytokines, the alarmin S100A8/A9 plays a crucial role in RA progression. We determined that CD64/*FCGR1A* expression correlates to *S100A8* and *S100A9* gene expression, S100A9 protein expression, and the secretion of S100A8/A9. S100A8/A9 serum levels are significantly correlated with biomarkers for RA including C-reactive protein, rheumatoid factor and erythrocyte sedimentation rate, and Disease Activity Score of 28 joints [17, 18]. They also seem to be a prognostic marker for radiographic damage and progression in cross-sectional and longitudinal studies [17, 19] that can be used to monitor response to treatment [18, 20–22]. In addition to a correlation with inflammatory marker expression, *FCGR1A* expression positively correlated to gene expression of collagenases (*MMP1* and *MMP13*), stromelysin-1 (*MMP3*), and gelatinase B (*MMP9*), but not to gelatinase A (*MMP2*) and the transmembrane MMP (*MMP14*). It was previously reported that inhibition of MMP9, but not MMP2, decreased the production of pro-inflammatory cytokines and suppressed RA synovial fibroblast-mediated cartilage degradation [23], although others reported that MMP2 expression is associated with bone erosion in RA [24]. The collagenases *MMP1* and *MMP3* strongly correlate to *FCGR1A* and are well-known for their role in collagen degradation and correlation to disease activity and radiographic damage in RA [25]. CD64 thus reflects the production of pro-inflammatory cytokines, S100A8/A9, and important and relevant MMPs in RA synovium, which are known to be involved in RA progression. This indicates that CD64 potentially is a good marker to image the extent and damaging potential of synovitis.

In RA the number of sublining macrophages is highly sensitive to change after effective treatment [1, 26]. Consequently, synovial CD64 expression may also serve as a novel biomarker to predict or monitor treatment response in RA. The upregulation of CD64 in RA synovium compared to healthy non-inflamed synovium and its association with the damaging potential of the synovitis makes CD64 a potentially useful imaging marker. Fueldner et al. found that a high level of synovitis in RA leads to increased synovial CD64 expression, which suggested that CD64 may be used as a sensitive biomarker [27]. Interestingly, in addition, CD64^+^ cells seem to be involved in RA progression. Selective removal of CD64^+^ cells from the synovial fluid or synovial explants of RA patients with CD64-immunotoxins reduced pro-inflammatory cytokine production [28, 29]. In an in vivo model of adjuvant arthritis in human CD64 transgenic rats, treatment with CD64-directed immunotoxin reduced inflammation and bone erosion [30]. CD64 likely plays a role in RA-associated inflammation as IgG-ICs are present in RA which drive RA pathology. Together, these studies suggest that CD64 is an important receptor for the induction of both inflammation and damage during RA, making it a good candidate for molecular imaging of the activation state and damaging potential of synovitis in RA.

Previous research already indicates that CD64 is responsive to anti-inflammatory treatments. Intra-articular glucocorticoid injection in RA joints downregulates CD64 expression [6]. In contrast, dexamethasone did not alter the expression of CD64 on monocytes from systemic lupus erythematosus (SLE) patients in vitro, while it did inhibit the upregulation of monocytic CD64 induced by IFNγ, IFNα, and IL-12 [15]. Interestingly, Matt et al. revealed that the amount of CD64 on monocytes of early RA patients discriminates responders from non-responders to methotrexate and prednisolone treatment [13]. Combined, these studies indicate that CD64 might be a good imaging marker to assess the response to anti-inflammatory or anti-rheumatic drugs in RA.

A good imaging marker that predicts the damaging potential of synovitis in RA would associate to the extent of synovitis and correlate to the expression of pro-inflammatory cytokines and pro-damaging factors. Moreover, it should be expressed in early disease and preferably a membrane-associated protein which facilitates imaging. We revealed that CD64 is associated with factors known to be involved in inflammation and damage in RA and verified its expression in early and late disease. Imaging modalities for other macrophage markers have been developed among others directed at folate receptors (FOLR). FOLRβ is expressed by hematopoietic myeloid cells, including monocytes [31], macrophages [32], and myeloid leukemia cells [33]. Using a publicly available RNA-sequence database from early RA synovium [34] (https://peac.hpc.qmul.ac.uk/), we determined that *FCGR1A* gene expression more strongly correlates to clinical parameters compared to *FOLR2*, the gene encoding FOLRβ (Supplemental Table 1). In the same dataset, we found that *FCGR1A* expression is higher in the lymphoid and myeloid pathotypes compared to the fibroid pathotype, which was not the case for *FOLR2* (Supplemental Table 2). This indicates that CD64 imaging may be of complementary value to FOLRβ imaging for use in the clinic compared for which a [18F]fluoro-polyethylene glycol (PEG)-folate positron emission tomography (PET) tracer was developed and successfully used in arthritic rats [35] and human RA patients [36]. In addition, Orange et al. revealed 3 distinct synovial subtypes (high inflammatory, low inflammatory, and mixed), *FCGR1A* expression was higher in the high inflammatory cluster [37]. Future studies, directly comparing the two, will be very informative with respect to the potential of both tracers in detecting specific pathotypes and early or destructive diseases. In addition, future studies should address the functioning of the CD64 tracer in different RA pathotypes. Imaging tracers, including PET tracer ^18^ F-fluoro-2-deoxy-D-glucose (^18^ F-FDG) or SPECT tracer ^99m^technetium-methylene diphosphonate (99mTc-MDP) are already in use in clinics to assess inflammation and bone damage in RA patients. These tracers have proven to be useful, however, they visualize general processes such as glucose metabolism and bone turnover and provide limited insight into the disease process [38–40].

In imaging studies, we aim to use the lowest possible protein dose that shows favorable retention in the target tissue. This is to prevent unwanted therapeutic effects from higher antibody doses. At 30 μg the specific uptake of the tracer was lower than at 10 μg, therefore we did not proceed with this higher dose group. Furthermore, in our study, the maximal achieved specific activity for the used antibodies is 1 MBq/μg antibody. Since USPECT imaging typically requires around 10 MBq of indium-111 for informative imaging within one hour it was not feasible to use 3 μg, despite its favorable distribution profile. Therefore, 10 μg/mouse was selected for the other experiments. At the chosen imaging interval, the isotype antibody showed considerable uptake in the synovium at this antibody dose. This can, however, largely be attributed to a much longer retention of the tracer in the circulation. Specific uptake, as denoted by the synovium-to-blood ratio, was much higher in the CD64-targeted antibody compared to the isotype control. This is also reflected by the clear demarcation of the implanted synovium on both the USPECT/CT scans and fluorescence images.

The goal of this study was to develop an imaging modality to detect the extent and characteristics of synovitis using CD64 as an imaging marker. We successfully developed such an imaging modality. Future proof of concept studies should be conducted to determine if CD64 also holds promise as a marker for the damaging potential in RA patients. Moreover, the next steps in the direction of developing a PET tracer (which has a higher resolution and sensitivity in a clinical setting) should be taken. Furthermore, it should be noted that CD64 is not exclusively expressed in the synovial tissue of RA patients. Neutrophil infiltration due to septic arthritis, or macrophage activation as the result of, e.g., osteoarthritis and spondyloarthropathies will most likely result in increased CD64 expression in the affected joint. Therefore, we believe that CD64 imaging should not be used as a diagnostic tool, but rather to collect additional information about the damaging potential of the synovitis after an individual has been diagnosed using the standard and well-established diagnostic criteria. Nevertheless, CD64 tracers could potentially be used to study other (chronic) inflammatory conditions in which CD64^+^ cells play a prominent role such as osteoarthritis, inflammatory bowel diseases [41] and SLE [42] once diagnosed. To this end, the SCID model could prove to be a valuable tool to bridge the gap between animal work and clinical trials.

## Conclusion

We revealed that the expression of *FCGR1A*/CD64 correlates to the gene expression of several pro-inflammatory cytokines, the alarmin S100A8/A9, and multiple tissue degrading enzymes, which are well-known for their involvement in RA progression. This indicates that CD64 is a good imaging marker for the extent and characteristics of synovitis. In our study, we developed an imaging modality which enables the detection of CD64. The use of molecular imaging to predict or determine response to treatment at the individual patient level might be beneficial for patient outcomes and is a considerable step towards tailored personal medicine.

### Supplementary Information

**Additional file 1: Supplemental table 1.** *FCGR1A* correlates better to clinical parameters of RA compared to *FOLR2. ***Supplemental table 2.** *FCGR1A* gene expression is different in various RA pathotypes. Supplemental methods. **Supplemental table 3.** RT-qPCR primer sequences. **Supplemental figure 1.** Flow chart showing the collection, processing and analysis procedure of early (yellow) and late-stage RA (blue; used for *in vitro* studies and red; used to set up the CD64 imaging modality) synovium. **Supplemental figure 2.** Immunohistochemical staining of CD64 from early-stage RA patients. Representative pictures of 10 early-stage RA patients included in this study are depicted and ordered from low (top) to high CD64 expression (bottom). Scale bar indicates 50 µm. **Supplemental figure 3.** Immunohistochemical staining of CD64 from late-stage RA patients. Representative pictures of 24 late-stage RA patients included in this study are depicted and ordered from low (top) to high CD64 expression (bottom). Scale bar represents 50 µm. **Supplemental figure 4.** Scoring system of synovial infiltration. **Supplemental figure 5.** Immunohistochemical staining of CD64 correlates with the staining intensity of CD64 measured with the Odyssey Clx imager. **Supplemental figure 6.** Anti-human CD64 antibody does not binds to murine CD64 on cultured macrophages. **Supplemental figure 7.** Dose escalation study of [^111^In]In-DTPA-IRDye 800CW anti-CD64 in SCID mice implanted with human RA synovium. **Supplemental figure 8.** USPECT-CT images of mice injected with [^111^In]In-DTPA-IRDye 800CW anti-CD64 or [^111^In]In-DTPA-IRDye 800CW isotype.

## Data Availability

Available upon request.

## Abbreviations

RA: Rheumatoid arthritis
MRI: Magnetic resonance imaging
FcγRI: Fc gamma receptor I
Ig: Immunoglobulin
IgG-ICs: IgG-containing immune complexes
DTPA: Diethylenetriamine penta-acetic acid
SCID: Severe combined immunodeficient
PBS: Phosphate-buffered saline
IR: Infrared
RT: Room temperature
ITC: Isothiocyanatobenzyl
DAS28: Disease Activity Score evaluated in 28 joints
IQR: Interquartile range
DMEM: Dulbecco’s modified Eagle’s medium
RNA: Ribonucleic acid
RT-qPCR: Real-time quantitative polymerase chain reaction
H&E: Hematoxylin and eosin
SPECT: Single-photon emission computed tomography
CT: Computed tomography
RPMI: Roswell Park Memorial Institute
CB17/lcr-Prkdcscid/Rj: CB17-SCID mice
FDR: False discovery rate
IL: Interleukin
TNF: Tumor necrosis factor
S100: S100 calcium-binding protein
IHC: Immunohistochemistry
MMPs: Matrix metalloproteases
TNFSF: TNF Superfamily Member
ACP: Acid phosphatase
CTSK: Cathepsin K
%ID: % Injected dose
SLE: Systemic lupus erythematosus
FOLR: Folate receptors
PEG: Polyethylene glycol
PET: Positron emission tomography
^18^F-FDG: ^18^F-fluoro-2-deoxy-D-glucose
99mTc-MDP: ^99M^technetium-methylene diphosphonate
