# Supplementary material for: CD64 as novel molecular imaging marker for the characterization of synovitis in rheumatoid arthritis
Source: Arthritis Res Ther. 2023 Aug 31;25:158. doi: 10.1186/s13075-023-03147-y (PMC10468866; doi:10.1186/s13075-023-03147-y)
Supplement: Supplementary file 1 — Additional file 1: Supplemental table 1. FCGR1A correlates better to clinical parameters of RA compared to FOLR2. Supplemental table 2. FCGR1A gene expression is different in various RA pathotypes. Supplemental methods. Supplemental table 3. RT-qPCR primer sequences. Supplemental figure 1. Flow chart showing the collection, processing and analysis procedure of early (yellow) and late-stage RA (blue; used for in vitro studies and red; used to set up the CD64 imaging modality) synovium. Supplemental figure 2. Immunohistochemical staining of CD64 from early-stage RA patients. Representative pictures of 10 early-stage RA patients included in this study are depicted and ordered from low (top) to high CD64 expression (bottom). Scale bar indicates 50 µm. Supplemental figure 3. Immunohistochemical staining of CD64 from late-stage RA patients. Representative pictures of 24 late-stage RA patients included in this study are depicted and ordered from low (top) to high CD64 expression (bottom). Scale bar represents 50 µm. Supplemental figure 4. Scoring system of synovial infiltration. Supplemental figure 5. Immunohistochemical staining of CD64 correlates with the staining intensity of CD64 measured with the Odyssey Clx imager. Supplemental figure 6. Anti-human CD64 antibody does not binds to murine CD64 on cultured macrophages. Supplemental figure 7. Dose escalation study of [111In]In-DTPA-IRDye 800CW anti-CD64 in SCID mice implanted with human RA synovium. Supplemental figure 8. USPECT-CT images of mice injected with [111In]In-DTPA-IRDye 800CW anti-CD64 or [111In]In-DTPA-IRDye 800CW isotype. [file 13075_2023_3147_MOESM1_ESM.docx]

**Supplemental information**

**Supplemental table 1: *FCGR1A* correlates better to clinical parameters of RA compared to *FOLR2***

|  | ***FCGR1A*** | | ***FOLR2*** | |
| --- | --- | --- | --- | --- |
| **Parameter** | **r** | ***P* adj** | **r** | ***P* adj** |
| ***CD3 (T cells)*** | 0.48 | ***0.00017*** | 0.22 | 0.089 |
| ***CD20 (B cells)*** | 0.57 | ***6.6x10^-7^*** | 0.25 | ***0.037*** |
| ***CD68L (Monocytes/Macrophages-lining)*** | 0.48 | ***0.0016*** | 0.28 | ***0.04*** |
| ***CD68SL (Monocytes/Macrophages-sublining)*** | 0.61 | ***5.6x10^-7^*** | 0.17 | 0.15 |
| ***CD138 (Plasma cells)*** | 0.51 | ***1.3x10^-5^*** | 0.24 | ***0.041*** |
| ***CCP*** | 0.065 | 0.53 | 0.03 | 0.26 |
| ***RF*** | 0.26 | 0.49 | 0.12 | 0.64 |
| ***CRP*** | 0.46 | ***0.0038*** | 0.026 | 0.53 |
| ***ESR*** | 0.45 | ***0.0018*** | 0.11 | 0.3 |
| ***DAS28-ESR*** | 0.41 | ***0.0069*** | 0.13 | 0.3 |
| ***DAS28-CRP*** | 0.47 | ***0.003*** | 0.077 | 0.51 |
| ***Tender joint counts*** | 0.20 | 0.57 | 0.026 | 0.82 |
| ***Swollen joint counts*** | 0.31 | 0.083 | 0.072 | 0.41 |
| ***VAS*** | 0.34 | 0.11 | 0.091 | 0.47 |
| ***HAQ-sum*** | 0.23 | 0.7 | 0.19 | 0.71 |
| ***US ST 12 max*** | 0.19 | 0.89 | 0.034 | 0.9 |
| ***US PD 12 max*** | 0.36 | 0.88 | 0.13 | 0.91 |
| ***US ST BJ*** | 0.40 | ***0.0059*** | 0.20 | 0.15 |
| ***US PD BJ*** | 0.32 | 0.052 | 0.089 | 0.42 |
| ***Total SHSS*** | 0.00067 | 0.67 | 0.0083 | 0.66 |

**Supplemental table 2: *FCGR1A* gene expression is different in various RA pathotypes**

| **Gene** | ***FCGR1A*** | | | ***FOLR2B*** | | |
| --- | --- | --- | --- | --- | --- | --- |
| **Pathotype** | **L** | **M** | **F** | **L** | **M** | **F** |
| **Maximum** | 11,83890 | 10,71153 | 10,04757 | 14,48546 | 14,20428 | 13,50564 |
| **Quartile 3** | 10,80277 | 10,04438 | 9,097228 | 13,00179 | 12,80311 | 12,91551 |
| **Median** | 10,22092 | 9,610602 | 8,26083 | 12,77856 | 12,55172 | 12,36105 |
| **Quartile 1** | 9,703631 | 9,135595 | 7,14349 | 12,48244 | 12,20467 | 12,15147 |
| **Minimum** | 8,327994 | 6,878021 | 6,484408 | 11,84882 | 11,96776 | 11,71149 |
| ***P* adj L *vs* M** | ***0,00209*** | | | 0,18 | | |
| ***P* adj L *vs* F** | ***3,17x10^-12^*** | | | 0,79 | | |
| ***P* adj M *vs* F** | ***0,00741*** | | | ***0,04*** | | |
| **Group** | L+M+ | | | not grouped | | |

L= lymphoid pathotype, M= myeloid pathotype and F= fibroid pathotype.

**Supplemental methods**

**RNA isolation, cDNA synthesis and RT-qPCR**

Freshly isolated synovial tissue was snap frozen and preserved in liquid nitrogen. Synovium was transferred into MagNA Lyser green beads tubes (Roche) containing RLT buffer (Qiagen) and homogenized using the MagNA Lyser Instrument (Roche Diagnostics). Total RNA was isolated using the RNeasy Kit (Qiagen) according to manufactory protocol. RNA quantity and purity was assessed with Nanodrop. RNA was treated with DNAse, reversed transcribed into complementary (c)DNA. The expression of a set of genes that encode for factors that are known to be involved in in RA pathology (pro-inflammatory cytokines, matrix degradation enzymes, and markers of bone remodelling) was measured with RT-qPCR using specific primers **(Supplemental table 3**) and SyBr green in the Applied Biosystems StepOnePlus RT-PCR System (Thermo Fisher Scientific). Expression levels were normalized to the average of two reference genes (glyceraldehyde 3-phosphate dehydrogenase (*GAPDH*) and ribosomal protein s27 (*RPS27*)) by calculating −ΔC_t_ = − (C_t_ gene of interest − C_t_ average *GAPDH* and *RPS27*).

**Supplemental table 3: RT-qPCR primer sequences**

| **Gene** | **Forward primer (5’-3’)** | **Reverse primer (5’-3’)** |
| --- | --- | --- |
| *FCGR1A* | CTCTGCTCCTTTGGGTTCCA | GCAAGGTTACGGTTTCCTCTTG |
| *IL1B* | TGGGTAATTTTTGGGATCTACACTCT | AATCTGTACCTGTCCTGCGTGTT |
| *TNFA* | TCTTCTCGAACCCCGAGTGA | CCTCTGATGGCACCACCAG |
| *IL6* | AGCCCACCGGGAACGA | GGACCGAAGGCGCTTGT |
| *IL8* | AGAAGTTTTTGAAGAGGGCTGAGA | CAGACCCACACAATACATGAAGTG |
| *S100A8* | AGGGATGACCTGAAGAAATTGCTA | GACGTCTGCACCCTTTTTCCT |
| *S100A9* | TCATCAACACCTTCCACCAATACT | GACCTTTTCATTCTTATTCTCCTTCTTG |
| *MMP1* | ACTGCCAAATGGGCTTGAAG | TTCCCTTTGAAAAACCGGACTT |
| *MMP2* | TCACTCCTGAGATCTGCAAACAG | TCACTCCTGAGATCTGCAAACAG |
| *MMP3* | GAGGCATCCACACCCTAGGTT | TCAGAAATGGCTGCATCGATT |
| *MMP9* | CTTCCAGTACCGAGAGAAAGCCTAT | CAGGACGGGAGCCCTAGTC |
| *MMP13* | ATTAAGGAGCATGGCGACTTCT | CCCAGGAGGAAAAGCATGAG |
| *MMP14* | TCACTCCTGAGATCTGCAAACAG | TCACTCCTGAGATCTGCAAACAG |
| *TNFSF11* | CAAGCTTGAAGCTCAGCCTTTT | TTATGGGAACCAGATGGGATG |
| *ACP5* | ACCATGACCACCTTGGCAAT | AGTTCCAGCGCTTGGAGATCT |
| *CTSK* | ACACCCACTGGGAGCTATGG | CGAGAGATTTCATCCACCTTGTT |
| *GAPDH* | ATCTTCTTTTGCGTCGCCAG | TTCCCCATGGTGTCTGAGC |
| *RPS27* | TGGCTGTCCTGAAATATTATAAGGT | CCCCAGCACCACATTCATCA |

**Preparation of synovial tissue-conditioned medium and S100A8/A9 measurements**

Synovial tissue-conditioned medium (CM) was obtained by culturing synovial tissue samples in RPMI supplemented with 10% fetal calf serum (FCS), 10.000 U/mL penicillin/streptomycin and 1% pyruvate for 24h. CM was centrifuged at 300 g for 5 min and supernatant was collected and stored at -80°C. The S100A8/A9 concentration in the CM was determined by an in house developed sandwich ELISA as described previously[1].

**Histology, immunohistochemistry, immunofluorescence and autoradiography**

From the OCT blocks 6 µm tissue sections were prepared with CryoStar NX70 (Thermo Fisher). Samples were mounted, packaged and stored at -80°C. Sections were used for histological analysis, CD64 (IHC and immunofluorescence) and S100A9 (immunofluorescence) quantification and for autoradiography (**Supplemental methods**).

**Immunohistochemistry**

Cryosections were thawed at room temperature (RT) for 30 min and fixed in cold acetone for 10 min. After blocking endogenous peroxidase with 1% H_2_O_2_ in phosphate-buffered saline (PBS), sections were stained with anti-CD64 antibody (Clone 10.1, Labned) or the respective isotype control (Dako). Afterwards, sections were incubated with a biotinylated secondary antibody followed by avidin-biotin complex peroxidase (VECTASTAIN Elite Kit; Vector Laboratories). Antibody binding was visualized using diaminobenzidine.

**Immunofluorescence**

Cryosections were thawed at RT, washed in water for 30 min and fixed in ice-cold acetone for 10 min. Sections were stained with anti-CD64 antibody (Labned), or anti-S100A9 (kindly provided by Dr. T. Vogl) or the respective isotype controls. Four consecutive sections were used for the staining. After washing in PBS, sections were incubated with IRDye 800CW-Goat anti-Mouse IgG or IRDye 800CW-Goat anti-rabbit (LICOR Bioscience). Subsequently, nuclear staining was performed with DRAQ5 (Biostatus) and sections were mounted with Fluor G mounting buffer (Thermo Fisher). Tissue slides were scanned with the Odyssey CLx Infrared Imaging System (LICOR Bioscience) and analyzed with Image studio Lite software v 5.2.5 (LICOR Bioscience). The DRAQ5 signal was used to manually draw the area of the synovium section of which the intensity of the CD64 , S100A9 or the respective isotype control signals were automatically quantified. The results were expressed as intensity/pixel calculated as background corrected signal/area.

**Histological analysis**

After scanning with the Odyssey CLx Infrared Imaging System the coverslips were unmounted with PBS and the sections were stained with hematoxylin and eosin (H&E) for histological analysis. Sub-lining immune cell infiltration was scored on blinded samples using an arbitrary score ranging from 0 to 3 (0: no infiltrating cells. 1: only mild infiltration mostly near the blood vessel and sub-lining. 2: moderate infiltration. 3: maximal infiltration, diffusely throughout the tissue) (**Supplemental Figure 4**)[2].

**Autoradiography**

Synovium cryosections were thawed at RT, fixed in cold acetone for 10 min and stained with [^111^In]In-DTPA-IRDye 800CW anti-CD64 or [^111^In]In-DTPA-IRDye 800CW isotype. After an extensive wash with PBS the sections were mounted in Fluor G mounting buffer (Thermo Fisher) and placed on a photostimulable phosphor plate. The plate was imaged after 1 week using photo-stimulated luminescence on a phosphor imager (Typhoon FLA 7000 phosphor imager, GE Healthcare). Thereafter, the sections were scanned in the Odyssey CLx Infrared Imaging System (LICOR Bioscience).

***In vitro* murine macrophage differentiation**

Total bone marrow cells from femurs of C57BL/6 mice were isolated, seeded in Nunc Up Cell Surface plates and differentiated towards macrophages in DMEM (Thermo Fisher Scientific), supplemented with 10% FCS, penicillin/streptomycin, pyruvate and 15 ng/mL of rmM-CSF (R&D systems). Medium was refreshed after 3 days. After 6 days, cells were stimulated with 20 ng/mL rmIFNγ and 100 ng/mL LPS for 24h. Cells were stained with an antibody directed at murine or human CD64 and measured with flowcytometry (see below).

**Flow cytometry staining and analysis**

Bone marrow-derived macrophages were washed with PBS, detached from the culture plate by incubation in PBS at 4°C and harvested. After incubation with Fc Block (2.4G2, BD Pharmingen), cells were incubated with Alexa Fluor 647-labeled anti-murine CD64 (clone X54-5/7.1, Cat No. 558539, BD Pharmingen) or unlabeled anti-human CD64 (Clone 10.1, Labned, 11-644-C100) followed by Alexa Fluor 488-labeled chicken anti-mouse secondary antibody (Invitrogen). Mouse IgG1κ was used as control for the human anti-CD64 antibody. Cells were washed with PBS, stained with fixable Viability Dye eFluor 450 (eBioscience) and fixed with 1% paraformaldehyde in PBS, followed by visualization using the Gallios fluorescence-activated cell sorting machine and analyzed using Kaluza 2.0 software (both from Beckman Coulter). For analysis, debris was excluded based on forward and side scatter, followed by the selection of single and viable cells. Subsequently, the mean fluorescent intensity (MFI) in the channel of interest was determined.

**Supplemental figures**

**
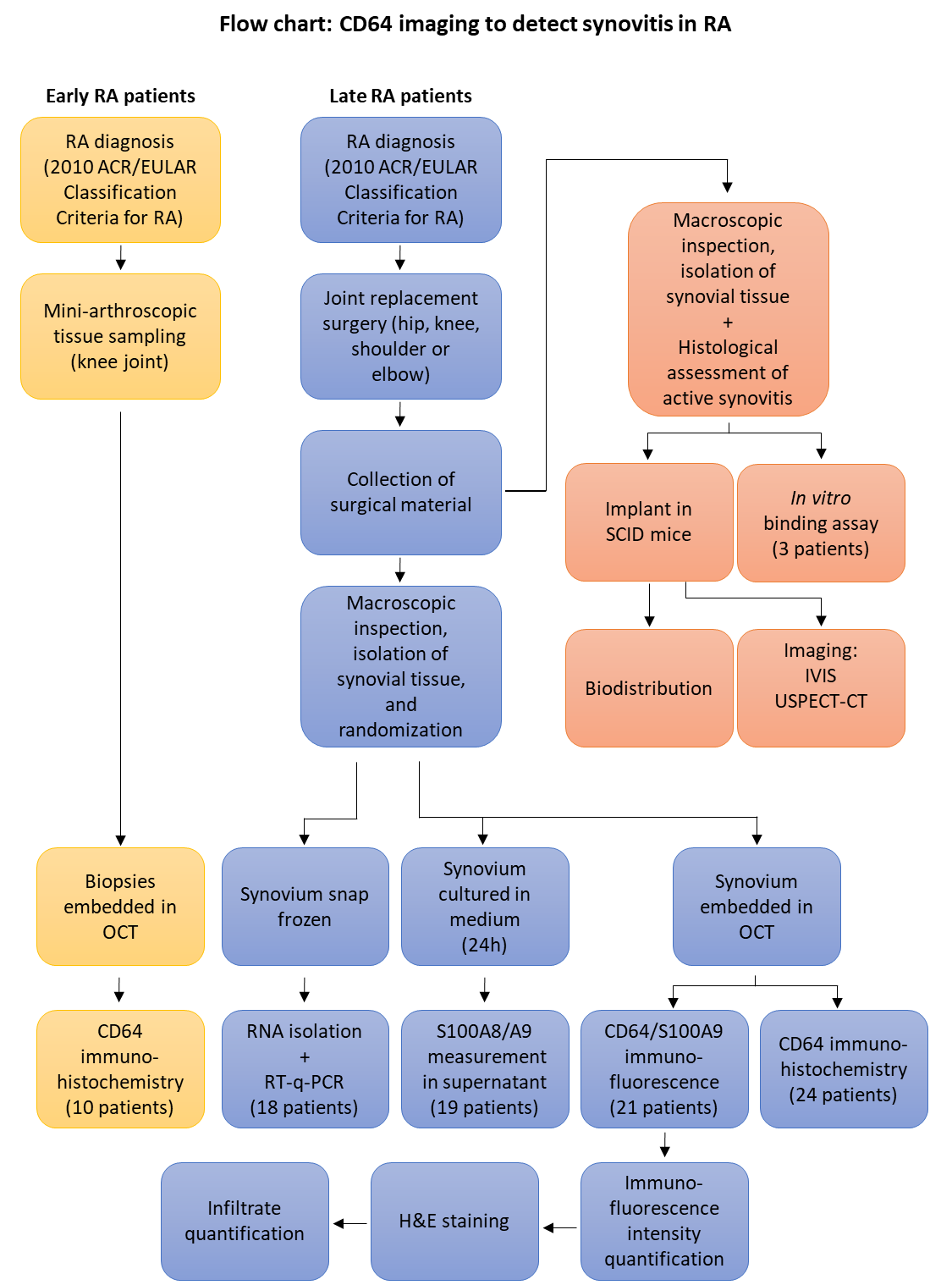
Supplemental figure 1:** Flow chart showing the collection, processing and analysis procedure of early (yellow) and late-stage RA (blue; used for *in vitro* studies and red; used to set up the CD64 imaging modality) synovium.


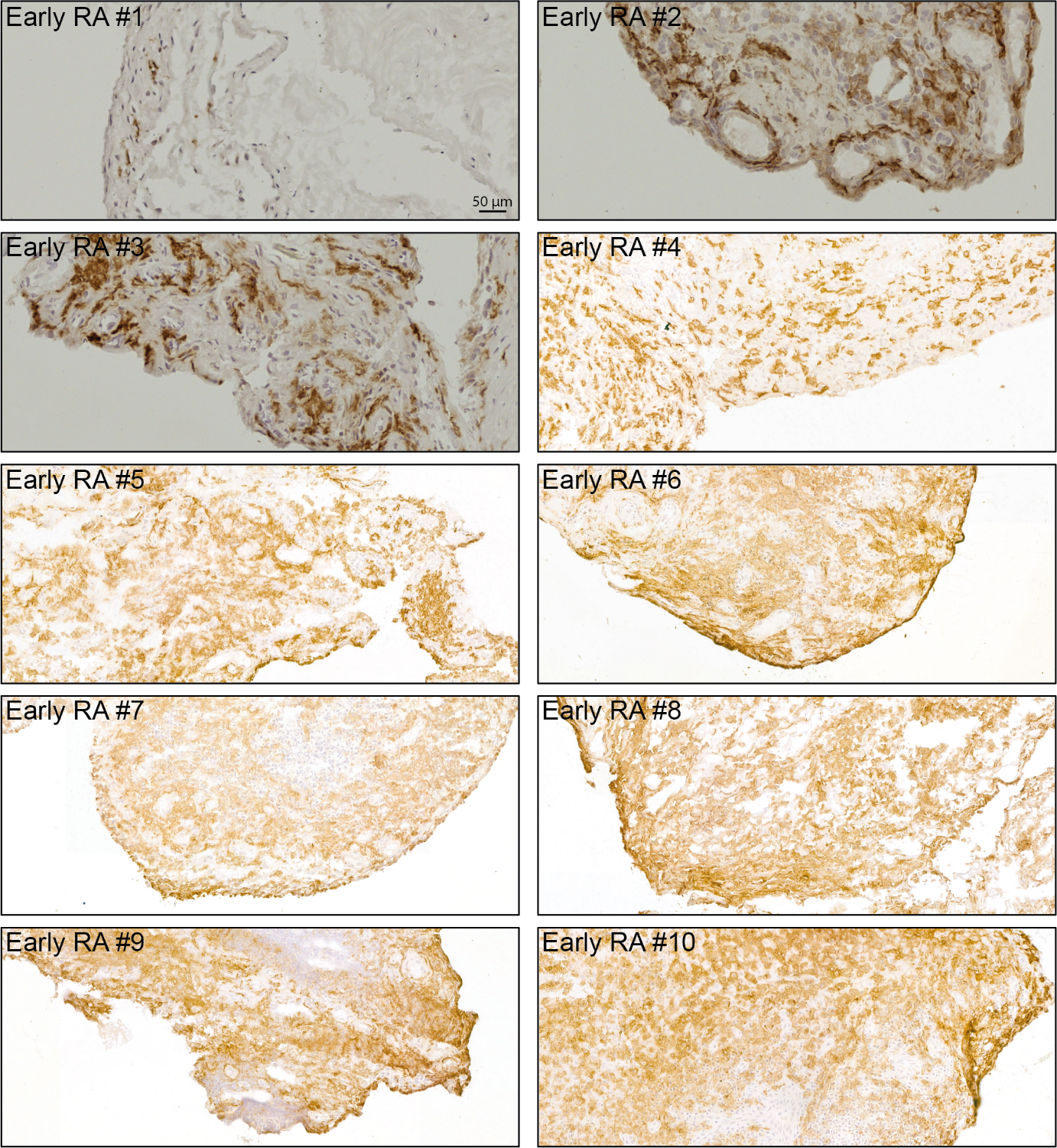


**Supplemental figure 2: Immunohistochemical staining of CD64 from early-stage RA patients.** Representative pictures of 10 early-stage RA patients included in this study are depicted and ordered from low (top) to high CD64 expression (bottom). Scale bar indicates 50 µm.


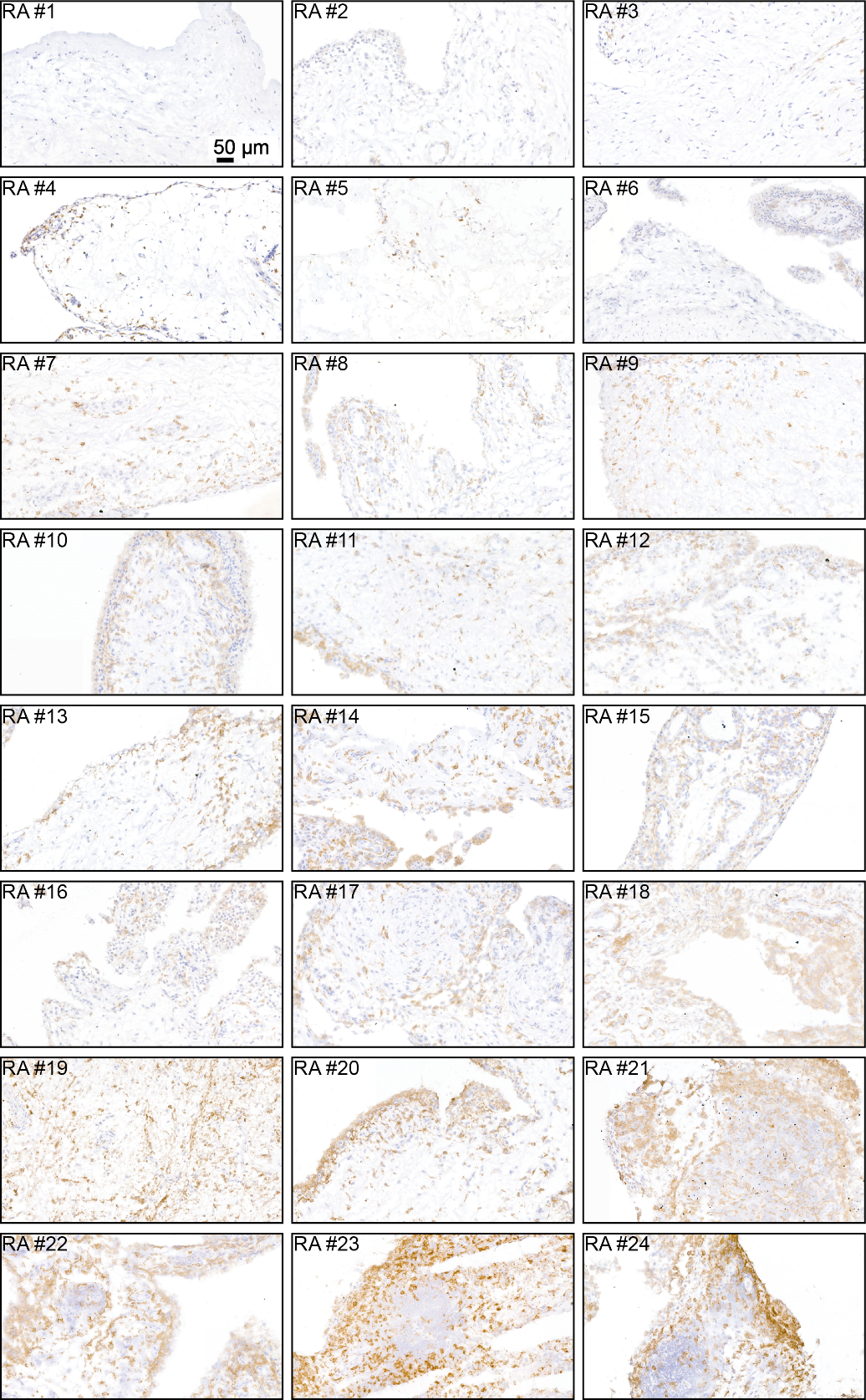


**Supplemental figure 3: Immunohistochemical staining of CD64 from late-stage RA patients.** Representative pictures of 24 late-stage RA patients included in this study are depicted and ordered from low (top) to high CD64 expression (bottom). Scale bar represents 50 µm.


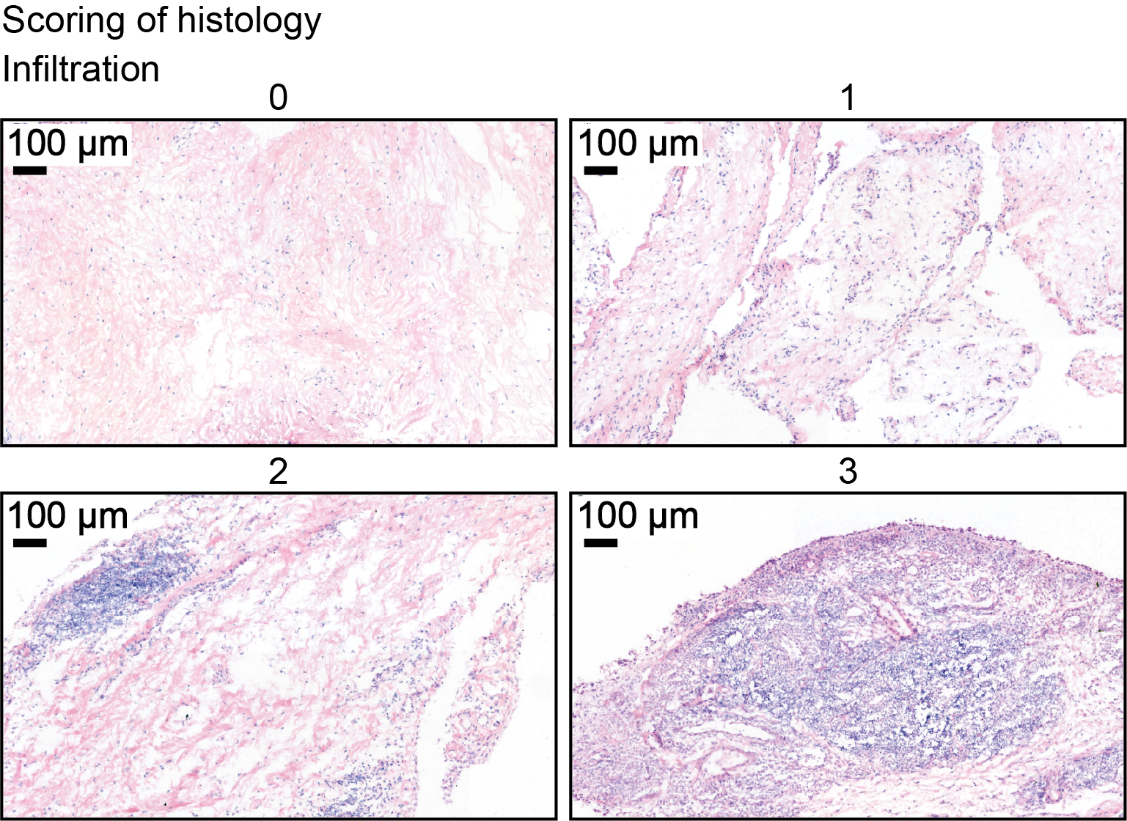


**Supplemental figure 4: scoring system of synovial infiltration.** Synovium inflammation was evaluated using an arbitrary score ranging from 0 to 3; 0: no infiltrating cells. 1: only mild infiltration, mostly near the blood vessel and sub-lining. 2: moderate infiltration. 3: maximal infiltration, diffusely throughout the tissue. Representative pictures showing the degree of infiltration ranging from 0 to 3. Original magnification 10x, scale bar indicates 100 µm.


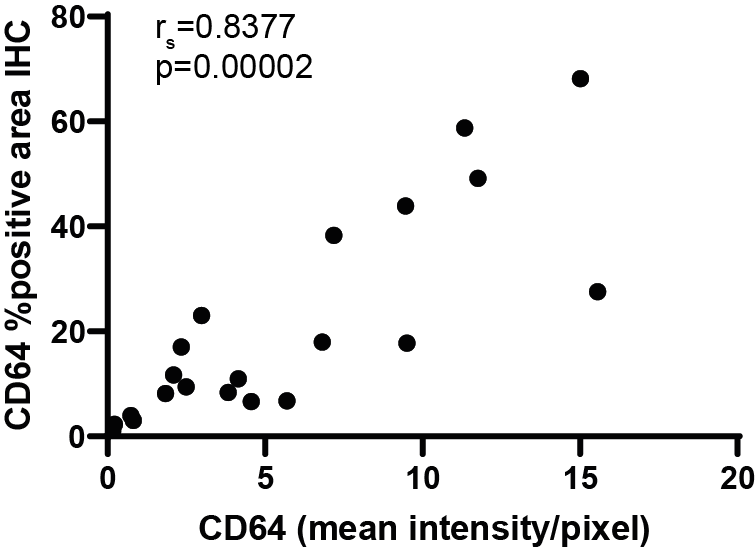


**Supplemental figure 5: Immunohistochemical staining of CD64 correlates with the staining intensity of CD64 measured with the Odyssey Clx imager.** Anti-CD64 antibodies were used to detect CD64 with IHC and fluorescent imaging using the Odyssey CLx imager. The % positive area of the IHC staining significantly correlates with the mean intensity/pixel from the fluorescent imaging. Each data point in the XY graph represents a value of 1 patient. n=21 patients, r_s_= Spearman’s rank correlation coefficient, IHC= immunohistochemistry.


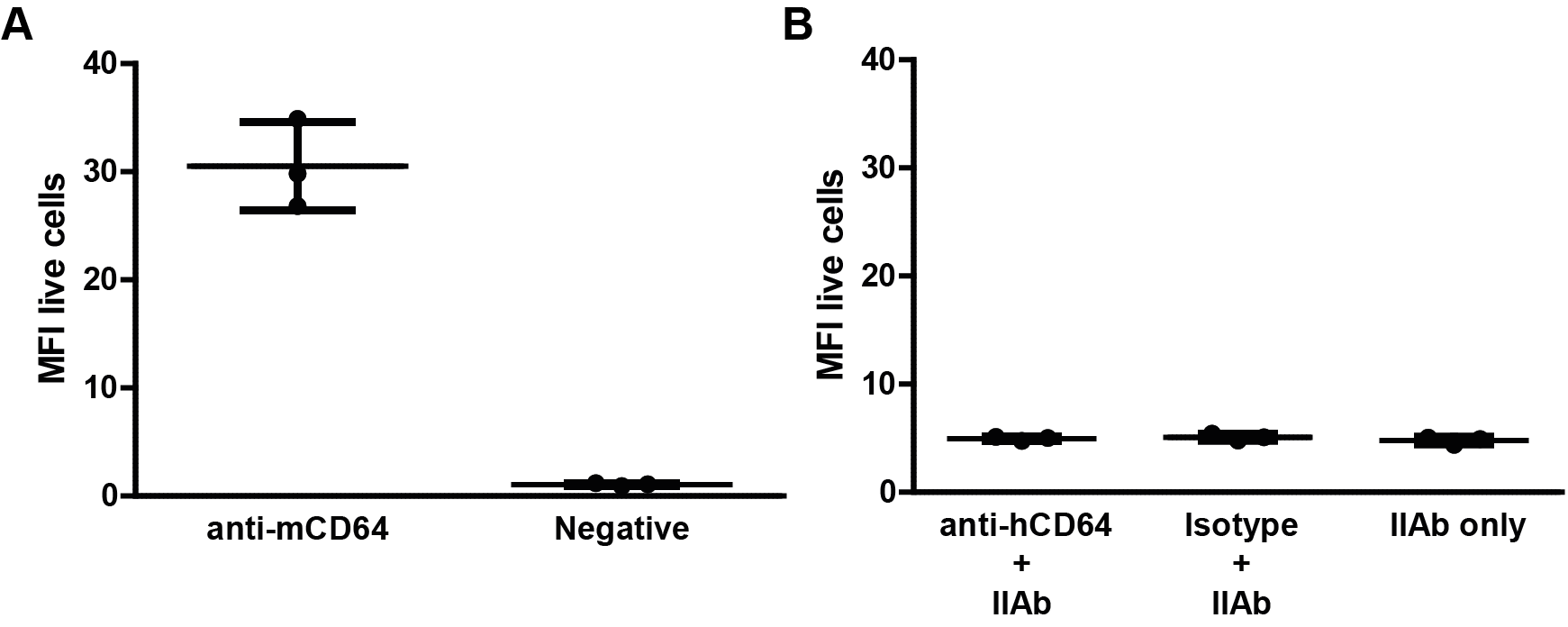


**Supplemental figure 6: Anti-human CD64 antibody does not binds to murine CD64 on cultured macrophages.** Murine macrophages were differentiated from bone marrow with rmM-CSF and polarized using rmIFNγ and LPS. (A) Flow cytometry staining using an anti-mouse CD64 confirmed the expression of CD64 on the differentiated polarized murine macrophages. Despite the expression of CD64 on the murine macrophages, (B) no binding was observed for the selected anti-human CD64 suggesting the specificity of the antibody for human CD64. Scatter plot showing mean±SD. MFI= mean fluorescent intensity.


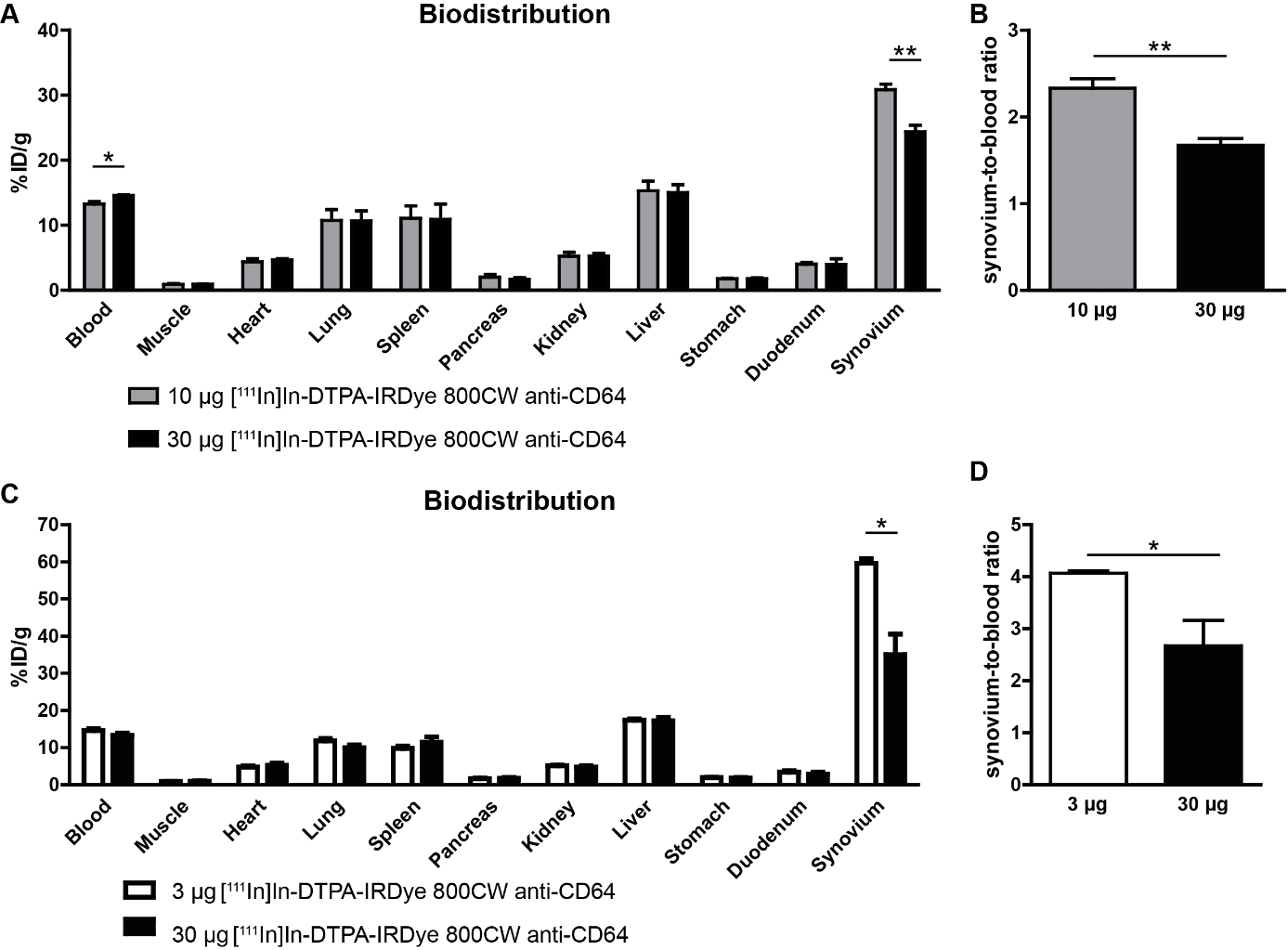


**Supplemental figure 7: Dose escalation study of [^111^In]In-DTPA-IRDye 800CW anti-CD64 in SCID mice implanted with human RA synovium.** SCID mice were subcutaneously implanted with human RA synovium obtained from patients undergoing joint replacement surgery. After at least 7 days from implantation mice were injected with (A) 10 (grey bars) and 30 (black bars) µg/mouse or (C) 3 (white bars) and 30 µg/mouse of [^111^In]In-DTPA-IRDye 800CW anti-CD64. After 48h from injection, biodistribution studies were conducted. (B, D) The synovium-to-blood ratio is higher for both 3 and 10 µg/mouse of anti-CD64 antibody compared to 30 µg/mouse. Bar graphs showing mean±SEM (n=3 mice/group). **p*<0.05, ***p*<0.01.


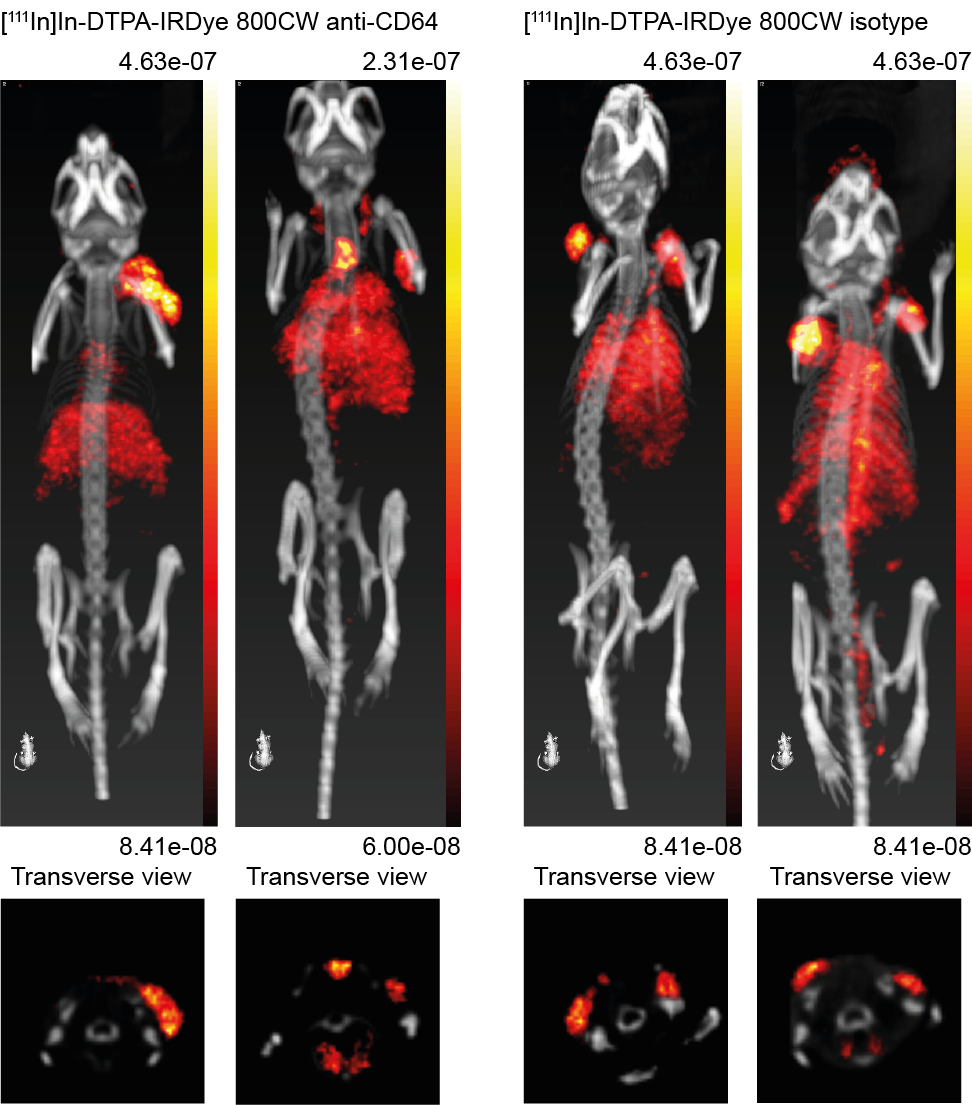


**Supplemental figure 8: USPECT-CT images of mice injected with [^111^In]In-DTPA-IRDye 800CW anti-CD64 or [^111^In]In-DTPA-IRDye 800CW isotype.** [^111^In]In-DTPA-IRDye 800CW anti-CD64 or [^111^In]In-DTPA-IRDye 800CW isotype (10 µg/mouse) were injected in SCID mice implanted with inflamed RA synovium. Representative USPECT-CT showing the uptake of [^111^In]In-DTPA-IRDye 800CW anti-CD64 (two left) or [^111^In]In-DTPA-IRDye 800CW isotype (two right) in the implanted synovium 48h after antibody injection (n=2/group).

**References**

1. Wulffraat, N.M., et al., Myeloid related protein 8 and 14 secretion reflects phagocyte activation and correlates with disease activity in juvenile idiopathic arthritis treated with autologous stem cell transplantation. Ann Rheum Dis, 2003. 62(3): p. 236-41.

2. Minten, M.J.M., et al., Exploring longitudinal associations of histologically assessed inflammation with symptoms and radiographic damage in knee osteoarthritis: combined results of three prospective cohort studies. Osteoarthritis Cartilage, 2019. 27(1): p. 71-79.
